# Usability and satisfaction of electronic medical records Interface and its relationship with work design among healthcare professionals at universal health insurance settings in Port Said, Egypt

**DOI:** 10.1016/j.hpopen.2026.100184

**Published:** 2026-09-29

**Authors:** Mervat Mahmoud Ali Elsangidy, Nesrine Saad Farrag, Celine Tabche, Salman Rawaf, Enas Elsheikh, Nesreen F. Ibrahim

**Affiliations:** a Department of Public Health, Faculty of Medicine, Port Said University, Bur Sa'id, Egypt; b School of Public Health, Faculty of Medicine, Imperial College London, UK

**Keywords:** Electronic medical records, Digital health, User satisfaction, Work design, Health system development, Public health

## Abstract

The effective use of electronic medical records (EMRs) has become increasingly vital to ensuring quality and safety in healthcare delivery in Egypt. Yet, user satisfaction and perceptions of usability often hinge on how work is structured within clinical settings. This study aimed to assess how healthcare professionals in Egypt, perceive the usability of EMR interface and how their satisfaction is shaped by the work design under the Universal Health Insurance (UHI) system.

**Methods:**

An analytical cross-sectional study was conducted among 532 healthcare professionals working in UHI-affiliated primary and secondary healthcare facilities. Data was collected using a structured questionnaire that combined the Questionnaire for User Interaction Satisfaction and Short Version of the Work Design. Statistical analysis was performed using non-parametric methods in SPSS.

**Results:**

Healthcare professionals reported an overall median EMR satisfaction score of 6.8. Screen design received the highest rating (median = 7.3), followed by terminology and system information and learning (both median = 7.1), while system capabilities (median = 6.6) and general impressions (median = 6.5) received lower ratings. The median overall work-design score was 3.7, with task significance and social support rated most favorably, while autonomy received relatively lower scores. A moderate positive correlation was observed between work design and EMR satisfaction (ρ = 0.48, *p* < 0.001). In bivariate analyses, gender and language used for EMR data entry were significantly associated with EMR satisfaction, while years of EMR experience were associated with work design perceptions. In the multivariable model, work design was the only independent predictor of overall EMR satisfaction (β = 0.47, *p* < 0.001).

**Conclusion:**

Improving system responsiveness and addressing work-design characteristics, particularly autonomy, social support and feedback from others, may support better EMR user experiences.

## Highlights

• Moderate EMR satisfaction linked to supportive work environments.
• Task design and social support strongly correlated with EMR satisfaction.
• Primary care staff reported better work design than hospital counterparts.
• Male and Arabic-language users showed higher EMR satisfaction levels.
• EMR experience improved perceptions of overall work design.

## 1 Introduction

The digital transformation of healthcare systems worldwide has led to the widespread adoption of electronic medical records (EMRs), which have become a central component of modern health information management. EMRs are developed to enhance the quality, safety, and efficiency of healthcare delivery by improving data accessibility, communication, and evidence-based decision-making. [1] While EMRs have demonstrated clear benefits, their implementation, particularly in low- and middle-income countries (LMICs), remains complex often hindered by infrastructural limitations, inconsistent usability, and limited engagement from frontline healthcare workers [2], [3].

In Egypt, the recent implementation of the Universal Health Insurance (UHI) system marks a significant step toward achieving universal health coverage. Port Said was selected as the pilot governorate for this reform, with EMR systems introduced in primary and secondary healthcare facilities affiliated with UHI. These digital systems aim to standardize clinical workflows, ensure accurate patient documentation, and enable long-term health data management [4], [5]. However, the true success of such initiatives depends not only on the availability of infrastructure or policy support but critically on the satisfaction and acceptance of EMR users, healthcare professionals who interact with these platforms daily [6].

In Port Said, the initial governorate for UHI implementation, EMR-enabled services were introduced across primary and hospital-based healthcare facilities. The digital system supports unified electronic patient records, clinical documentation and management, electronic prescriptions and referrals, radiology archiving, revenue-cycle management, and health-information exchange across healthcare and insurance entities. The system is operated within the national UHI framework through the Egypt Healthcare Authority (EHA) and eHealth, with digital health services supported by standardized health information and coding systems, including ICD-11. Detailed information regarding the underlying vendor configuration and server or cloud architecture was not available to the study team.

Usability is a key determinant of EMR adoption and is commonly defined as the degree to which users can effectively, efficiently, and satisfactorily achieve goals within a given system [7]. Poor interface design may result in user frustration, workflow disruption, and even adverse patient outcomes [8], [9]. Conversely, user-centered EMR designs have been associated with improved productivity, greater satisfaction, and enhanced integration into routine clinical tasks [10].

Importantly, EMR usability does not exist in a vacuum. The broader work environment, including how tasks are structured and supported, plays a critical role in shaping user experiences. Work design elements, such as task autonomy, feedback, social support, and cognitive load, can significantly influence how healthcare providers interact with digital systems and perceive their value [11], [12].

Despite increasing EMR use across Egypt's public health sector, research exploring user satisfaction and its linkage to work design remains scarce. Existing studies have predominantly addressed technical performance or policy-level barriers, often overlooking the real-world experiences of healthcare professionals in clinical settings [3]. This knowledge gap limits the ability of decision-makers to implement user-centered improvements or tailor training and support efforts effectively.

Accordingly, this study aimed to: [1] assess healthcare professionals' satisfaction with and perceived usability of the EMR interface; [2] evaluate key work-design characteristics among healthcare professionals using EMRs; and [3] examine the association between EMR satisfaction and work design within UHI-affiliated healthcare facilities in Port Said.

## 2 Methods

### 2.1 Study design

This study employed an analytical cross-sectional design to evaluate EMR usability and satisfaction, assess work-design characteristics, and examine the association between EMR satisfaction and work design among healthcare professionals.

### 2.2 Study setting

The study was conducted between October, November, and early December 2023 across multiple healthcare facilities affiliated with the UHI system in Port Said, Egypt. The settings included both primary care units and secondary general hospitals. Specifically, the primary care level encompassed 36 family medicine units and centers, while the secondary care level comprised 5 general hospitals. Facilities not operating under the UHI framework or still relying on paper-based systems were excluded from the study [13].

### 2.3 Study population

The target population comprised healthcare professionals working in UHI-affiliated facilities in Port Said who used EMR systems as part of their routine work. Participants included physicians, nurses, pharmacists, technicians, and other healthcare professional groups represented in the study sample. The ‘other’ category included physiotherapists and other professional groups with small cell sizes. The study population was restricted to healthcare professionals represented in the clinical and allied-health occupational categories reported. Duration of EMR use was recorded as a study variable and varied among participants. Eligibility was based on employment in a UHI-affiliated healthcare facility and routine use of the EMR system as part of professional practice.

### 2.4 Sampling procedure

A non-probability sampling approach was used. Convenience sampling was applied for participant recruitment, while quota sampling by facility type was used to ensure representation from both primary and secondary healthcare settings. Recruitment targets were approximately 7–10 participants per primary care unit or center and 40–50 participants per hospital. Information on the total number of eligible healthcare professionals at each facility was not available; therefore, the proportionality of recruitment quotas to facility staffing levels could not be assessed. The manuscript has been revised to clarify the combined convenience and quota sampling approach. Detailed participant-level recruitment procedures were not available in the study records and therefore have not been inferred. Based on the available recruitment records, the overall response rate was approximately 80%.

The sample size was determined using a single-proportion formula [14], based on a previously reported EMR satisfaction rate of 40% from a study conducted among physicians in a major government hospital in Saudi Arabia [15]. Using this reference point, with a 95% confidence level and a 5% margin of error, the minimum required sample size was calculated to be 369 participants. The minimum sample size was estimated using the single-proportion formula, n = Z^2^p(1 − p)/d^2^, assuming *p* = 0.40, a 95% confidence level and d = 0.05, yielding a minimum required sample of 369 participants. To enhance representativeness and statistical power, the final sample included in this study was 532 healthcare professionals from both primary and secondary healthcare settings.

Because participants were recruited within healthcare facilities, potential facility-level clustering was recognized as a feature of the sampling design. However, a design effect was not incorporated into the original sample-size calculation, and the analysis did not explicitly model facility-level clustering. This is therefore considered a methodological limitation of the study. Consequently, standard errors and confidence estimates may not fully account for within-facility correlation.

### 2.5 Data collection tool

Data was collected using a structured, self-administered questionnaire composed of three sections:

Section 1**:** Sociodemographic and professional characteristics (e.g., age, gender, job title, years of EMR experience, computer skills, language proficiency).

Section 2**:** EMR usability and satisfaction, measured using the Questionnaire for User Interaction Satisfaction (QUIS) covering five key dimensions: screen design, terminology/system information, learning, system capabilities, and general impressions. Each item was rated on a 9-point Likert scale [16].

Section 3**:** Work design characteristics assessed using the Short Version of the Work Design Questionnaire (WDQ—S), which evaluates autonomy, task significance, feedback, information processing, skill variety, social support, and work context using a 5-point Likert scale [17].

Domain and overall scores were analyzed as continuous scale scores and are reported as medians with interquartile ranges because the distributions were non-normal. The instruments were translated into Arabic and subsequently back-translated into English to assess linguistic and conceptual consistency. A pilot test involving 144 participants was conducted prior to the main study. No modifications were made to the questionnaire items, Arabic translation, questionnaire layout, administration procedures, or study protocol following the pilot test. Accordingly, the pilot participants were retained in the final analytical sample.

### 2.6 Validity and reliability

Content validity was assessed by a panel of three experts in public health and health informatics. The experts evaluated the relevance and clarity of the questionnaire items, and the Scale-Level Content Validity Index (S-CVI) was calculated. Language accuracy of the Arabic versions was also assessed by bilingual experts. For QUIS, the S-CVI values were 1.00 for relevance and 0.93 for clarity, while language accuracy was 81.8%. For WDQ—S, the corresponding values were 1.00, 0.97, and 92.5%, respectively. Any incorrect or unclear phrases identified during the validation process were modified accordingly.

Internal consistency was assessed using Cronbach's alpha, yielding α = 0.982 for QUIS and α = 0.949 for WDQ—S. The very high internal consistency observed for QUIS was interpreted cautiously, as such a high coefficient may partly reflect redundancy among some items. Construct validity of the Arabic versions was not formally assessed using exploratory or confirmatory factor analysis; therefore, the psychometric evaluation in this study focused on content validity, language accuracy, and internal consistency.

### 2.7 Data management and statistical analysis

All data were processed and analyzed using SPSS version 26. The dataset was first cleaned and checked for completeness and outliers. The Kolmogorov-Smirnov test was used to assess normality. Given that most variables were not normally distributed, non-parametric tests were applied:

**Descriptive statistics:** medians and interquartile ranges (IQR) for continuous variables; frequencies and percentages for categorical variables.

**Inferential statistics:** Mann-Whitney *U* test and Kruskal-Wallis tests were used for group comparisons. Spearman's rank correlation coefficient (ρ) was used to assess associations between EMR satisfaction and work design variables. A *p*-value less than 0.05 was considered statistically significant.

A multiple linear regression analysis was subsequently performed to identify independent predictors of overall EMR satisfaction. The dependent variable was the overall EMR satisfaction score. Independent variables included overall work design score, years of EMR experience, duration of EMR use per day, job category, language used for EMR data entry, and workplace setting. Categorical variables were dummy-coded, with ‘Others’ as the reference category for job, use of both Arabic and English as the reference category for language, and hospital as the reference category for workplace. Unstandardized regression coefficients (B), standard errors, standardized coefficients (β), *p*-values and variance inflation factors (VIFs) were reported. Statistical significance was set at *p* < 0.05.

**Missing data handling:** Questionnaires with missing responses in items required to calculate the primary study outcomes (QUIS and WDQ—S) were excluded from the corresponding analysis. Missing responses in sociodemographic and work-related variables did not result in exclusion of the questionnaire; instead, analyses involving these variables were conducted using the available non-missing observations. No statistical imputation was performed for missing values.

The number of observations included in each analysis therefore varied according to the availability of complete data for the variables involved, and the corresponding denominators are reported in the tables. A formal statistical assessment of the missing-data mechanism was not undertaken; therefore, no assumption was made that the data were missing completely at random, missing at random, or missing not at random.

### 2.8 Ethical considerations

Ethical approval was obtained from the Institutional Review Board (IRB) at Port Said University. Authorization was also secured by the Universal Health Insurance Authority. All participants provided informed consent prior to data collection. Confidentiality and anonymity were maintained throughout, and all data were securely stored and accessible only to IRB-approved personnel. Participants were informed of their right to withdraw at any point without any repercussions.

## 3 Results

A total of 532 healthcare professionals participated in the study. The median age of respondents was 30.50 years (IQR = 12), with a predominantly female sample (64.70%). The majority were employed in primary care units (62.20%), and most were married (60.50%) and residing within Port Said (71.30%). A small proportion reported having a disability (0.90%) or a chronic disease (10.20%) (Table 1).

**Table 1:** Sociodemographic characteristics of participants.

Sociodemographic characteristics	Median (IQR)	
Age (*n* = 426)	30.50(12)	
Frequency (percentage)		
Gender (*n* = 524)	Male	185 (35.30)
	Female	339 (64.70)
Working place	Units	331 (62.20)
	Hospitals	201 (37.80)
Marital status (*n* = 521)	Single	183 (35.10)
	Married	315 (60.50)
	Divorced	20 (3.80)
	Widowed	3 (0.60)
Permanent residency (*n* = 527)	Inside Port Said	376 (71.30)
	Outside Port Said	151 (28.70)
Suffering from any disability	Yes	5 (0.90)
	No	527 (99.10)
Suffering from any chronic diseases	Yes	54 (10.20)
	No	475 (89.30)
	Not sure	3 (0.50)

Note: The total sample size was 532. Missing responses were identified for age (*n* = 106), gender (*n* = 8), marital status (*n* = 11), and permanent residency (*n* = 5). No missing responses were identified for working place, disability, or chronic diseases. Percentages are calculated based on the available non-missing responses for each variable.

Nurses comprised the largest proportion of respondents (41.20%), followed by physicians (26%), pharmacists (14.50%), and technicians (14.30%). Most participants hold a bachelor's degree (48.70%) or an institute-level qualification (31.70%). The median duration of EMR use was 4 years, with a median of 7 h/day and 24 days/month.

The findings indicated that nearly half of the participants (48.10%) used a combination of Arabic and English when entering data into the electronic medical record system, while approximately one-third (33.64%) relied solely on Arabic. More than half (56.39%) rated their English proficiency as very good to excellent, and a similar proportion (54.13%) reported a high level of computer competency. By contrast, only a small minority had international exposure, with 5.82% having previously worked abroad and 1.87% having pursued studies abroad (Table 2).

**Table 2:** Professional characteristics of participants.

Professional characteristics	Frequency (percentage)	
Job (*n* = 531)	Physician	138 (26.00)
	Nursing	219 (41.20)
	Technician	76 (14.30)
	Pharmacist	77 (14.50)
	Others	21 (4.00)
Scientific degree (*n* = 530)	Secondary school	52 (9.80)
	Institute	168 (31.70)
	Bachelor	258 (48.70)
	Master	45 (8.50)
	Doctorate	6 (1.10)
	Fellowship	1 (0.20)
Language in entering medical records	Arabic	179 (33.64)
	English	97 (18.23)
	Both	256 (48.12)
Proficiency in English language	Weak to very weak	44 (8.27)
	Good	188 (35.34)
	Very good to excellent	300 (56.39)
Computer proficiency	Weak to very weak	34 (6.39)
	Good	210 (39.47)
	Very good to excellent	288 (54.13)
Working outside country	Yes	31 (5.82)
	No	501 (94.17)
Studying outside country	Yes	10 (1.87)
	No	522 (98.12)


Median (IQR)	
Duration of EMR use (years) (*n* = 391)	4 (3)
Daily EMR use (hours/day) (hours/day) (*n* = 455)	7 h (6)
EMR use (days/month) (*n* = 445)	24 days (11)

Note: The total sample size was 532. Missing responses were identified for job (*n* = 1), scientific degree (*n* = 2), years of dealing with medical records (*n* = 141), hours of dealing with medical records per day (*n* = 77), and days of dealing with medical records per month (*n* = 87). No missing responses were identified for the remaining variables. Percentages are calculated based on the available non-missing responses for each variable. The ‘Others’ category includes physiotherapists and other professional groups with small cell sizes.

Healthcare professionals reported an overall median EMR satisfaction score of 6.82 (IQR 2.11) on the 1–9 scale. They were generally pleased with how the system looked and how easy it was to navigate, especially praising the screen design, terminology, and how straightforward it was to learn, all scoring above 7. Many pointed out that the system's speed, the way it handled feedback and error messages, and the lack of advanced features or quick shortcuts made daily tasks more challenging (Table 3).

**Table 3:** User interaction satisfaction with EMR.

All satisfaction domains	median (IQR)
1. Screen	7.29 (1.87)
2. Terminology and system information	7.14 (2.29)
3. Learning	7.05 (2.26)
4. System capabilities	6.55 (2.70)
5. General impressions	6.50 (2.90)
Overall Satisfaction with EMR	6.82 (2.11)

The median overall work-design score was 3.63 (IQR 0.93). Task significance and social support had the highest median scores (4.00), while autonomy had a lower median score of 3.33. Contextual characteristics had a median score of 3.58, with physical demands and work conditions both scoring 3.66 (Table 4).

**Table 4:** Work design domain and subscale scores.

Work design subscales	median (IQR)
1. Task characteristics	3.66 (1.08)
1.1. Autonomy	3.33 (1.33)
1.2. Task Significance	4.00 (1.33)
1.3. Feedback from Job	3.66 (1.33)
2. Knowledge Characteristics	3.66 (1.17)
2.1. Information Processing	3.66 (1.33)
2.2. Skill Variety	3.66 (1.00)
3. Social Characteristics	3.66 (1.17)
3.1. Social Support	4 (1.33)
3.2. Feedback from Others	3.66 (1.33)
4. Contextual Characteristics	3.58 (1)
4.1. Physical Demands	3.66 (1.00)
4.2. Work Conditions	3.66 (1.33)
Total work design assessment	3.63 (0.93)

A moderate positive correlation was observed between overall EMR satisfaction and total work design score (ρ = 0.48, *p* < 0.001). The strongest associations were observed for task characteristics (ρ = 0.49) and social characteristics (ρ = 0.43). These findings indicate that more favorable perceptions of work design were associated with greater EMR satisfaction, although the cross-sectional design does not permit causal inference (Table 5).

**Table 5:** Correlation between Work Design Subscales and EMR Overall Satisfaction.

Task characteristics	EMR overall satisfaction ρ (*P*-value)
	0.49 (<0.001)
Knowledge Characteristics	0.34 (<0.001)
Social Characteristics	0.43 (<0.001)
Contextual Characteristics	0.35 (<0.001)
Total work design assessment	0.48 (<0.001)

Cell content: Spearman correlation(P-Value).

Bivariate analyses identified statistically significant differences in EMR satisfaction by gender and language used for EMR data entry. Female participants reported a higher median satisfaction score than male participants (6.72 vs 6.47; *p* = 0.02). Satisfaction also differed according to language used for EMR entry (overall *p* = 0.01), with Arabic-only users reporting a median score of 6.75, bilingual users 6.68, and English-only users 6.27. No statistically significant differences were observed across job categories (*p* = 0.23), workplace, marital status, residency, international work or study experience, disability status, or chronic disease status (Table 6, Table 7).

**Table 6:** Sociodemographic & Professional Determinants for EMR Overall Satisfaction.

^OBJ^Median (IQR)			P-value
Working place	Units	6.64 (2.09)	0.81^@^
	Hospitals	6.62 (2.15)	
Gender (*n* = 524)	Male	6.47 (2.08)	0.02^@^
	Female	6.72 (2.18)	
Marital status (*n* = 521)	Single	6.55 (2.26)	0.71^#^
	Married	6.69 (2.05)	
	Divorced	6.38 (2.61)	
	Widowed	7.20 (0.00)	
Residency(*n* = 527)	Inside Port Said	6.64 (2.1)	0.87^@^
	Outside Port Said	6.62 (2.46)	
Job (*n* = 531)	Technician	6.75 (1.77)	0.23^#^
	Nursing	6.72 (2.07)	
	Pharmacist	6.36 (2.08)	
	Physician	6.55 (2.17)	
	
	Others	6.93 (3.05)	
Language in entering medical records	Arabic^a^	6.75 (2)	0.01^#^
	English^b^	6.27 (1.74)	0.01^&ab^
	Both^c^	6.68 (2.13)	0.55^&ac^
			0.01^&bc^
Working outside country	Yes	6.74 (1.93)	0.81^@^
	No	6.62 (2.15)	
Studying outside country	Yes	6.52 (2.63)	0.87^@^
	No	6.63 (2.13)	
Suffering from any disability	Yes	6.59 (2.81)	0.83^@^
	No	6.63 (2.11)	
Suffering from any chronic diseases	Yes	6.40 (2.26)	0.36^#^
	No	6.65 (2.14)	
	Not sure	7.04 (0.00)	

Kruskal-Wallis Test results (#), otherwise Mann-Whitney Test was used (@).

Note: The total sample size was 532. Missing responses were identified for gender (*n* = 8), marital status (*n* = 11), residency (*n* = 5), and job (*n* = 1). No missing responses were identified for the remaining variables. Median and IQR were calculated based on the available non-missing observations for each variable.

**Table 7:** Correlation between Sociodemographic and professional characteristics of participants and EMR Overall Satisfaction.

Age	EMR overall satisfaction ρ (P-value)
	−0.04 (0.41)
Scientific degree	−0.07 (0.10)
Daily EMR use (hours/day)	0.08 (0.10)
EMR use (days/month)	0.02 (0.67)
Duration of EMR use (years)	0.03 (0.41)

Cell content: Spearman correlation(P-Value).

In bivariate analyses, healthcare professionals working in primary care units reported higher median work design scores than those working in hospitals (3.70 vs 3.54; *p* = 0.01). Years of EMR experience showed a weak positive correlation with work-design score (ρ = 0.15, *p* = 0.003). Age, gender, marital status, scientific degree, job category, daily EMR use, and monthly EMR use were not significantly associated with work design perceptions (See Table 8, Table 9, Table 10).

**Table 8:** Sociodemographic and Professional Determinants of Work Design.

		Median (IQR)	P-value
Working place	Units	3.70 (0.83)	0.01^@^
	Hospitals	3.54 (0.98)	
Gender (*n* = 524)	Male	3.55 (0.97)	0.08^@^
	Female	3.69 (0.93)	
Marital status (*n* = 521)	Single	3.65 (0.97)	0.49^#^
	Married	3.62 (0.91)	
	Divorced	3.40 (1.10)	
	Widowed	3.94 (0.81)	
Permanent residency (*n* = 527)	Inside Port Said	3.64 (0.92)	0.41^@^
	Outside Port Said	3.59 (0.95)	
Job (*n* = 531)	Physician	3.50 (1.01)	0.25^#^
	Nursing	3.66 (1.04)	
	Technician	3.49 (0.94)	
	Pharmacist	3.50 (0.75)	
	Others	3.77 (0.63)	

Kruskal-Wallis Test results (#), otherwise Mann-Whitney Test was used (@).

Note: The total sample size was 532. Missing responses were identified for gender (*n* = 8), marital status (*n* = 11), permanent residency (*n* = 5), and job (*n* = 1). No missing responses were identified for the working place. Median and IQR were calculated based on the available non-missing observations for each variable.

**Table 9:** Correlation between sociodemographic and professional characteristics of participants and work design.

Age	Work design ρ (P-value)
	0.01 (0.72)
Scientific degree	−0.07 (0.09)
Duration of EMR use (years)	0.15 (0.003)
Daily EMR use (hours/day)	−0.00 (0.95)
EMR use (days/month)	0.07 (0.11)

Cell content: Spearman correlation(P-Value).

**Table 10:** Multiple linear regression predicting overall satisfaction.

Predictor	B	SE	β	t	p	VIF
Work design	0.98	0.09	0.47	9.91	<0.001	1.05
Years of experience	−0.01	0.04	−0.01	−0.373	0.71	1.11
Duration of dealing with medical records/day	0.00	0.02	0.00	0.09	0.92	1.01
Job – Technician	−0.51	0.44	−0.11	−1.16	0.24	4.21
Job – Nursing	−0.50	0.41	−0.17	−1.22	0.22	8.71
Job – Pharmacist	−0.77	0.45	−0.17	−1.71	0.08	4.82
Job – Physician	−0.45	0.43	−0.13	−1.05	0.29	7.79
Language – Arabic	0.10	0.16	0.03	0.66	0.51	1.27
Language – English	−0.01	0.19	−0.00	−0.07	0.94	1.21
Workplace – Unit	−0.09	0.15	−0.03	−0.61	0.53	1.09

**Model fit: *R* = 0.49, R**^**2**^ **= 0.24, Adjusted R**^**2**^ **= 0.22, F (10.345) = 10.82, *p* < 0.001.** Reference categories were Others for job, both for language used in entering medical records, and Hospital for workplace.

The multiple linear regression model was statistically significant, F (10,345) = 10.82, *p* < 0.001, explaining 23.9% of the variance in overall EMR satisfaction (R^2^ = 0.24; adjusted R^2^ = 0.22). Work design was the only statistically significant independent predictor of overall EMR satisfaction (B = 0.98, β = 0.47, *p* < 0.001). Years of EMR experience, duration of EMR use per day, job category, language used for entering medical records and workplace setting were not statistically significant after adjustment (all *p* > 0.05).

## 4 Discussion

This study found that healthcare professionals in UHI-affiliated facilities in Port Said reported a moderate level of satisfaction with their EMR system. Users appreciated the clear screen design, intuitive terminology, and how easy it was to get started. Nevertheless, they reported concerns regarding system speed, responsiveness, and limited advanced functionality. Similar concerns have been reported among physicians using EMRs in Saudi Arabia, where slow system performance and inadequate feedback mechanisms were associated with poorer user experiences and workflow disruption [15]. These findings suggest that favorable interface design alone may be insufficient when system responsiveness and functionality do not adequately support clinical workflows.

The positive ratings in screen design and learning experience echo findings from a study in the UAE, where physicians in primary care centers emphasized how much easier their work became when EMRs offered simple navigation and visually organized information [18]. However, as seen in both our study and others, when EMRs lack depth in advanced features or fail to provide clear error messages, users tend to disengage or limit their use to basic functions [19]. This highlights a common gap: systems are often built for functionality but fall short in supporting long-term, efficient use.

Turning to work design, participants in our study felt their roles were meaningful and valued by the support they received from colleagues. This aligns with research from Canada and Poland, where healthcare workers consistently cited task significance and strong social ties as key reasons for job satisfaction even in environments with limited resources [20], [21]. Meaningful work and social support may be associated with more favorable perceptions of the work environment.

However, low scores in autonomy reflect a recurring issue in healthcare worldwide. A study from Saudi Arabia, conducted during the COVID-19 pandemic, found that healthcare workers operating under rigid hierarchies and strict protocols reported high stress levels and burnout due to limited decision-making power [22]. These findings highlight the importance of balancing team support with appropriate professional autonomy.

The strong correlation we found between EMR satisfaction and work design is not surprising. Studies in Spain and other countries have shown that when healthcare environments encourage collaboration and provide clear task structures, staff are more likely to embrace digital systems [23], [24]. These findings support the view that successful EMR implementation depends on alignment between system usability and the organizational context in which the technology is used. Successful EMR implementation appears to depend on alignment between system usability and the organizational context in which the technology is used.

These findings have practical implications for the continued expansion of the UHI system in Egypt. At the national level, EMR procurement and system evaluation should incorporate explicit usability and user-centered design requirements, including system responsiveness, clear feedback mechanisms, ease of learning, and compatibility with clinical workflows, as these factors are recognized as important dimensions of safe and effective health information system use [10], [11], [12]. From an infrastructure perspective, investment decisions should consider not only system availability but also the technical capacity required to maintain responsive and reliable EMR performance as implementation expands across facilities. At the organizational level, clinical governance and implementation frameworks should incorporate structured user feedback, role-sensitive training, and opportunities for greater autonomy and team-based support, reflecting the observed association between work design and EMR satisfaction in the present study. Such an approach may help the UHI system move beyond basic digitalization toward sustainable, user-centered implementation across different healthcare settings in Egypt.

Regional evidence also supports these findings. Neirat et al. reported perceived benefits of EMR implementation at Al-Razi Hospital in Palestine alongside barriers related to workload, staff training, computer availability and organizational support. This regional experience similarly highlights the importance of considering both system usability and organizational conditions during EMR implementation [25].

Bivariate analyses showed differences in EMR satisfaction according to gender and language used for data entry. Female participants reported a higher median satisfaction score than male participants. Arabic-only users reported a slightly higher median satisfaction score than bilingual users, while English-only users reported the lowest median score [26]. However, neither gender nor language remained statistically significant for predictors of overall satisfaction in the multivariable regression model. These bivariate differences should therefore be interpreted cautiously and may reflect interface familiarity, documentation practices, professional role or other unmeasured contextual factors rather than independent effects of gender or language.

Interestingly, factors like age, education, or years of EMR experience did not significantly impact satisfaction, something that recent research supports. As EMRs become more widespread, system design and workplace culture seem to outweigh personal demographics in determining user experience [27], [28].

In short, this study highlights that EMR satisfaction is associated with both system usability and the organization of work. Sustainable implementation may therefore require attention to technical performance, workflow design, professional autonomy and team support.

### 4.1 Limitations

The study acknowledges several limitations that may affect interpretation and generalizability. First, the use of a convenience sampling method may have introduced selection bias, limiting representativeness. Second, although no modifications were made to the questionnaire items, Arabic translation, questionnaire layout, administration procedures, or study protocol following the pilot test, the 144 pilot participants were retained in the final analytical sample. Therefore, pooling the pilot and main-study participants remains a potential methodological limitation. Third, reliance on self-reported questionnaires raises the possibility of recall or social desirability bias. Although multivariable regression was undertaken for overall EMR satisfaction, residual confounding from measured and unmeasured factors cannot be excluded. In addition, a separate multivariable model was not undertaken for work design; therefore, factors associated with work design should be interpreted as unadjusted associations. Fourth, the study's focus on one governorate (Port Said) under the UHIS pilot restricts applicability to other regions, rural areas, or private healthcare settings. Fifth, while quantitative measures provided structured insights, the absence of qualitative data limited understanding of deeper user experiences and contextual challenges. Lastly, unmeasured external factors such as digital competence, workload, burnout, and institutional support were not assessed, though evidence suggests they significantly influence EMR usability and staff satisfaction. Substantial missingness was present for selected variables, particularly duration of EMR use, which may have reduced precision in analyses involving these variables. Some occupational groups also contained very small numbers and were therefore combined into an “Others” category for inferential analyses; although this improved analytical stability, it may have obscured profession-specific differences in EMR usability and work design. Some methodological details were not available in the study records used for the present revision, including exact item-level scoring and reverse-scoring procedures, detailed translator roles, the sample used to calculate internal-consistency coefficients, instrument permission procedures, minimum prior EMR-use requirements, and participant-level recruitment procedures. The available study records did not permit confirmation of whether every eligible facility contributed participants or the precise stage at which facilities using paper-based records were excluded. These limitations highlight the need for more representative sampling, longitudinal and mixed methods design, and inclusion of broader organizational and individual variables in future research.

## 5 Conclusion

This study explored healthcare professionals' experiences with EMRs in Port Said and the influence of their work environment. Participants reported comparatively favorable scores for screen design, terminology and system information, and learning, with lower scores for system capabilities and general impressions. Work-design scores indicated comparatively high task significance and social support and lower autonomy. Work environment perceptions were similarly mixed: professionals found their roles meaningful and appreciated peer support but reported limited autonomy. A moderate positive association was observed between EMR satisfaction and work design, with work design remaining the only statistically significant independent predictor of overall satisfaction in the multivariable model. Although gender and language used for EMR data entry were associated with satisfaction in bivariate analyses, these associations did not persist following adjustment. Improving system responsiveness alongside supportive work design may therefore contribute to more sustainable and user-centered EMR implementation.

### 5.1 Recommendations

Based on the study findings, priority should be given to improving EMR responsiveness, system capabilities and feedback mechanisms, alongside organizational approaches that support appropriate professional autonomy and social support. Language accessibility and role-specific interface requirements may also warrant consideration during future system refinement. Broader interventions, including targeted training and profession-specific support, should be evaluated in future implementation studies before their effectiveness is assumed. These measures may contribute to improved user experience and more sustainable EMR implementation.

## CRediT authorship contribution statement

**Mervat Mahmoud Ali Elsangidy:** Writing – review & editing, Writing – original draft, Validation, Project administration, Methodology, Investigation, Formal analysis, Conceptualization. **Nesrine Saad Farrag:** Writing – review & editing, Methodology, Data curation. **Celine Tabche:** Writing – review & editing, Validation, Supervision, Methodology, Formal analysis. **Salman Rawaf:** Writing – review & editing. **Enas Elsheikh:** Writing – review & editing, Validation, Supervision, Conceptualization. **Nesreen F. Ibrahim:** Writing – review & editing, Visualization, Supervision.

## Clinical trial number

Not applicable.

## Ethical statement

This study was conducted in accordance with ethical standards and approved by the appropriate institutional review board. Participation was voluntary, and informed consent was obtained from all respondents. Ethical approval was obtained from the Institutional Review Board (IRB) at Port Said University.

## Funding

This research received no external funding.

## Declaration of competing interest

The authors declare no conflicts of interest related to this study.

## Data Availability

The datasets generated and analyzed during this study are available from the corresponding author upon reasonable request.
